# Effect of Position and Structure of the Terminal Moieties in the Side Group on the Liquid Crystal Alignment Behavior of Polystyrene Derivatives

**DOI:** 10.3390/polym13162822

**Published:** 2021-08-22

**Authors:** DaEun Yang, Kyutae Seo, Hyo Kang

**Affiliations:** BK-21 Four Graduate Program, Department of Chemical Engineering, Dong-A University, Busan 49315, Korea; 1830133@donga.ac.kr (D.Y.); kyutae@donga.ac.kr (K.S.)

**Keywords:** liquid crystal, alignment, polystyrene, terminal moiety, structure–property relationship

## Abstract

We synthesized a series of polystyrene derivatives containing various side groups, such as the 4-(*tert*-butyl)-phenoxymethyl, 3-(*tert*-butyl)-phenoxymethyl, 2-(*tert*-butyl)-phenoxymethyl, 4-cumyl-phenoxymethyl, and 4-trityl-phenoxymethyl groups, through a polymer modification reaction to examine the liquid crystal (LC) alignment of these derivatives. In general, the vertical LC alignment on polymer films can be affected by the position and structure of the terminal moiety of the polymer side group. For example, the LC cells fabricated with 4-(*tert*-butyl)-phenoxymethyl-substituted polystyrene having a *tert-*butyl moiety as a *para*-type attachment to the phenoxy groups of the polystyrene derivatives exhibited vertical LC alignment, whereas the LC cells prepared from 3-(*tert*-butyl)- and 2-(*tert*-butyl)-phenoxymethyl-substituted polystyrene films exhibited planar LC alignment. In addition, the LC cells fabricated from 4-cumyl- and 4-trityl-phenoxymethyl-substituted polystyrene films with additional phenyl rings in the side groups exhibited planar LC alignment, in contrast to the LC alignment of the (*tert*-butyl)-phenoxymethyl-substituted polystyrene series. The vertical LC orientation was well correlated with the surface energy of these polymer films. For example, vertical LC orientation, which mainly originates due to the nonpolar tertiary carbon moiety having bulky groups, was observed when the surface energy of the polymer was lower than 36.6 mJ/m^2^.

## 1. Introduction

Liquid crystals (LCs), which consist of elongated organic molecules with an uneven charge distribution along their dipoles, are materials with properties between those of ordinary liquids and three-dimensional solids [1,2,3]. LCs have been studied in elementary research and for the development of commercial applications owing to their exceptional anisotropic physicochemical characteristics. Based on the factors that impart the liquid crystalline properties, LCs can be classified into two generic categories: thermotropic and lyotropic mesophases [4,5]. Lyotropic LCs, consisting of amphiphilic molecules, can be subdivided into three phases: lamellar, hexagonal, and cubic phases. The transition among these three phases can be triggered spontaneously, depending on the water content of the aqueous solution. The unique structures of LC molecules, as well as the physicochemical properties of the lyotropic LC systems, render them potential candidates for pharmaceutical applications, such as drug delivery carriers and several other applications [6,7,8,9,10]. For example, the cubic phase has been investigated extensively for drug delivery systems. In addition, the cubic microstructure of the lyotropic LCs provides a release matrix for active materials of varying sizes and polarities owing to the dual polar–nonpolar nature [11,12,13,14]. Thermotropic LCs exhibit different liquid crystal phases as a function of temperature. Thermotropic LCs with a rod-like shape can be categorized into two groups, namely, nematic LCs and smectic LCs, based on the extent of the positional ordering of LC molecules. Nematic LCs have a characteristic long-range orientational order along the direction of the **n** vector, which is known as the director. In addition, the anisotropic physicochemical properties of the nematic LC molecules, such as refractive index and dielectric tensor, could be utilized in electro–optical devices such as displays and sensors by ensuring an appropriate orientation of LC molecules. This is because the nematic LC molecules are susceptible to external stimuli such as electric and/or magnetic field [15]. However, an incomplete orientation of LC molecules, such as disclination faults by the flow effect of LC injection, is observed in devices in which the order parameter of the surface orientation or the anchoring energy is low. The uniform/substantial orientation of the thermotropic LC molecules such as the nematic LCs plays an essential role in electro–optical applications. For example, the vertical orientation of nematic LCs, wherein the director of the nematic LCs is oriented vertically to the surface of the substrate, has been studied for sensor applications because of its susceptibility to small perturbations and binding events [16,17,18,19], which can be observed by optical apparatus such as a polarized optical microscope [20,21]. Orientation of LC molecules can be influenced by the anisotropic characteristics of the surface using numerous contact and noncontact methods such as mechanical rubbing, lithography, stretching, polarized ultraviolet radiation, and ion beam treatment [22,23,24,25,26,27,28,29]. Among these, mechanical rubbing on polymer surfaces is the most widely used contact method to obtain a uniform orientation of LC molecules. This is because the method is rapid and operationally simple [30,31]. Films of polyimide derivatives have been generically employed as LC orientation layers using the rubbing technique because these polymer films allow considerably stable LC orientations [32,33]. However, hard-baking is needed to produce appropriately oriented polyimide layers. The hard-baking temperature of conventional polyimide films is generally 200 °C or higher, which is too high for manufacturing flexible devices from plastic materials [34,35]. In addition, problems with negative effects have been encountered after the rubbing process; these include dust generation, physical damage, and electrostatic charge on the surface of the orientation layers [36,37,38]. Noncontact methods for the orientation of LC molecules have been investigated to overcome the drawbacks of the rubbing method. Photoalignment is a promising noncontact orientation technology for realizing next-generation LC display applications such as flexible displays, because of the inherent advantages of this method, such as cleanliness, lack of restrictions with respect to surface morphology, and suitability for large glass substrates. Polymers bearing various photoreactive functional groups for photoisomerization, photodimerization, and photodegradation have been studied as photoalignment layers [39,40,41]. The orientation of LC molecules on polymer films depends on the chemical composition of these films [42]. For example, polystyrene derivatives with long alkyl or fluoroalkyl groups have been developed for application in noncontact methods to achieve a vertical orientation of LC molecules on substrates. This is because the long alkyl or fluoroalkyl groups on the polystyrene layer result in low surface energy owing to the steric effects of these groups on the polymer film surface. Owing to the steric repulsion and/or the interactions between LC molecules and surfaces, the surface energy of the polymer films and the molecular orientation of the polymers are the decisive factors in obtaining a vertical LC orientation [43,44,45,46,47,48,49]. In previous studies, the vertical orientation of LC molecules in LC cells fabricated with PS derivatives substituted with natural extracts, such as capsaicin [47], eugenol [48], vanillin [49], oryzanol [50], and tocopherol [51], was observed when the substituent ratio was greater than 60 mol%. This is due to the long alkyl groups of the natural extracts, which are related to the low surface energy owing to the steric effect of the alkyl groups on the polymer film surface. In addition, the vertical alignment of the LC cells fabricated with PS derivatives having LC precursors in side chains, such as 4-(*trans*-4-ethylcyclohexyl)phenol [52], ethyl-*p*-hydroxybenzoate [53], and 4-ethyloxyphenol [54], was observed in spite of low substituent ratio from 20 mol%. In this case, it was found that the similarity between the chemical structure of the alignment layer and the LC molecule is a key factor in improving the LC alignment characteristics and inducing a stable alignment behavior. 

**Figure 1:**
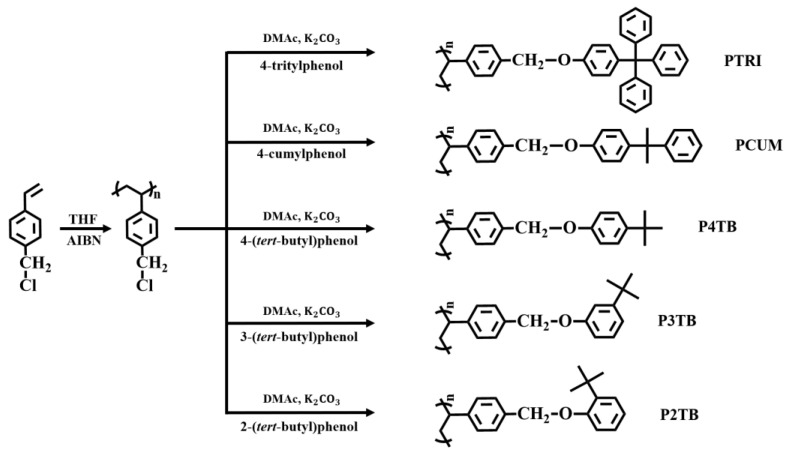
Synthetic routes of PTRI, PCUM, P2TB, P3TB, and P4TB films.

In this study, we synthesized a series of polystyrene derivatives having #-(*tert*-butyl)-phenoxymethyl (#TBs) units in the side groups (P#TBs); # indicates the position of the *tert*-butyl group with respect to the oxymethyl group in disubstituted benzenes (Figure 1). We also synthesized polystyrene derivatives having 4-cumyl-phenoxymethyl (PCUM) and 4-trityl-phenoxymethyl (PTRI) moieties in the side groups; these groups have an additional phenyl ring in the terminal moieties, in contrast to P#TBs. LC alignment according to the position and structure of the terminal moiety in the side group of the polystyrene derivatives was investigated. The bulk and surface properties of these polymers and the optical characteristics of LC cells fabricated with these polymer films were studied.

## 2. Materials and Methods

### 2.1. Materials

4-Chloromethylstyrene, 4-cumylphenol, and potassium carbonate were purchased from Aldrich Chemical Co. (Seoul, Republic of Korea). 4-Tritylphenol, 4-(*tert*-butyl)phenol, and 3-(*tert*-butyl)phenol were obtained from Tokyo Chemical Industry (Tokyo, Japan). 2-(*Tert*-butyl)phenol was purchased from Alfa Aesar (Lancashire, UK). Nematic LC, MLC-6608 (*n*_e_ = 1.559, *n*_o_ = 1.476, and ∆*ε* = −4.2, where *n*_e_, *n*_o_, and ∆*ε* are the ordinary refractive index, extraordinary refractive index, and dielectric anisotropy, respectively), was purchased from Merck Co. (Seoul, Republic of Korea). Ethanol and *N,N*′-dimethylacetamide (DMAc) were dried over molecular sieves (4 Å). 4-Chloromethylstyrene was purified by column chromatography on silica gel using hexane as the eluent to remove inhibitors (*tert*-butylcatechol and nitroparaffin) and any impurities. Tetrahydrofuran (THF) was dried under reflux over sodium and benzophenone and then distilled. Poly(4-chloromethylstyrene) (PCMS) was synthesized by conventional free radical polymerization of 4-chloromethylstyrene with 2,2′-azobisisobutyronitrile (AIBN) under a nitrogen atmosphere. AIBN (Daejung Chemicals & Metals Co., Siheung, Republic of Korea) was purified by crystallization from methanol and was used as the initiator. All other reagents and solvents were used as received. 

### 2.2. Preparation of #-(Tert-butyl)-phenoxymethyl-Substituted Polystyrene (P#TB)

The following procedure was used to synthesize all the #-(*tert*-butyl)-phenoxymethyl-substituted polystyrenes (# is 4, 3, or 2). The synthesis of 4-(*tert*-butyl)-phenoxymethyl-substituted polystyrene (P4TB) is discussed as a representative example. A mixture of 4-(*tert*-butyl)phenol (0.44 g, 2.95 mmol, 150 mol% relative to PCMS) and potassium carbonate (0.50 g, 3.55 mmol) in DMAc (30 mL) was heated to 75 °C. A solution of PCMS (0.30 g, 1.97 mmol) in DMAc (20 mL) was added to the mixture and magnetically stirred at 70 °C for 24 h under a nitrogen atmosphere. The solution mixture was cooled to room temperature and poured into methanol to obtain a white precipitate. The precipitate was further purified by reprecipitation of the DMAc solution in methanol several times, followed by washing with hot methanol to remove any residual salts and potassium carbonate. After drying overnight under vacuum, P4TB was obtained in a yield of over 80%. Other #-(*tert*-butyl)-phenoxymethyl-substituted polystyrenes having tertiary butyl substituents (P#TB) at different positions were synthesized using a similar procedure, except that 3-(*tert*-butyl)phenol or 2-(*tert*-butyl)phenol was used as the reactant. For example, P3TB and P2TB, where the numbers indicate the position of the *tert*-butyl group containing monomeric units in the polymer, were prepared with 3-(*tert*-butyl)phenol (0.44 g, 2.95 mmol, 150 mol% relative to PCMS) and 2-(*tert*-butyl)phenol (0.44 g, 2.95 mmol, 150 mol% relative to PCMS), respectively. Comparison of the integrated peak areas of the oxymethyl and phenyl groups revealed that the degree (%) of substitution from chloromethyl to oxymethyl group was ~100%.

P4TB ^1^H NMR (400 MHz, CDCl_3_, *δ*/ppm): *δ* = 0.9–1.5 (-*CH*_2_-*CH*-Ph-CH_2_-O-, -Ph-C*(CH*_3_*)*_3_, 12H), 4.6–5.0 (-Ph-*CH*_2_-O-Ph-, 2H), 6.2–7.4 (-CH_2_-CH-*Ph*-CH_2_-O-*Ph*-C(CH_3_)_3_, 8H).

P3TB ^1^H NMR (400 MHz, CDCl_3_, *δ*/ppm): *δ* = 1.2–1.5 (-*CH*_2_-*CH*-Ph-CH_2_-O-, -Ph-C*(CH*_3_*)*_3_, 12H), 4.7–5.0 (-Ph-*CH*_2_-O-Ph-, 2H), 6.1–7.3 (-CH_2_-CH-*Ph*-CH_2_-O-*Ph*-C(CH_3_)_3_, 8H).

P2TB ^1^H NMR (400 MHz, CDCl_3_, *δ*/ppm): *δ* = 1.2–1.5 (-*CH*_2_-*CH*-Ph-CH_2_-O-, -Ph-C*(CH*_3_*)*_3_, 12H), 4.7–5.2 (-Ph-*CH*_2_-O-Ph-, 2H), 6.2–7.4 (-CH_2_-CH-*Ph*-CH_2_-O-*Ph*-C(CH_3_)_3_, 8H).

### 2.3. Preparation of 4-Cumyl-phenoxymethyl-Substituted Polystyrene (PCUM)

PCUM was synthesized using a procedure similar to that used for preparing P4TB, except that 4-cumylphenol (0.63 g, 2.95 mmol, 150 mol% relative to PCMS) was used instead of 4-(*tert*-butyl)phenol. The product was obtained in a yield of over 80%. Comparison of the integrated peak areas of the oxymethyl peak in the range 4.6–5.0 ppm and phenyl peaks in the range 6.2–7.5 ppm revealed that the degree (%) of substitution from the chloromethyl to oxymethyl group was ~100%.

PCUM ^1^H NMR (400 MHz, CDCl_3_, *δ*/ppm): *δ* = 1.0–1.5 (-*CH*_2_-*CH*-Ph-CH_2_-O-, 3H), 1.5–1.7 (-O-Ph-C*(CH*_3_*)*_2_-Ph, 6H), 4.6–5.0 (-CH_2_-CH-Ph-*CH*_2_-O-, 2H), 6.2–7.5 (-CH_2_-CH-*Ph*-CH_2_-O-*Ph*-C(CH_3_)_2_-*Ph*, 13H).

### 2.4. Preparation of 4-Trityl-phenoxymethyl-Substituted Polystyrene (PTRI)

4-Trityl-phenoxymethyl-substituted polystyrene (PTRI) was synthesized using a procedure similar to that used for preparing P4TB, except that 4-tritylphenol (0.99 g, 2.95 mmol, 150 mol% relative to PCMS) was used instead of 4-(*tert*-butyl)phenol. The product was obtained in a yield of over 80%. Comparison of the integrated peak areas of the oxymethyl peak in the range 4.6–5.0 ppm and phenyl peaks in the range 6.2–7.5 ppm revealed that the degree (%) of substitution from chloromethyl to oxymethyl group was ~100%.

PTRI ^1^H NMR (400 MHz, CDCl_3_, *δ*/ppm): *δ* = 0.9–1.5 (-*CH*_2_-*CH*-Ph-CH_2_-O-, 3H), 4.6–5.0 (-CH_2_-CH-Ph-*CH*_2_-O-, 2H), 6.2–7.5 (-CH_2_-CH-*Ph*-CH_2_-O-*Ph*-C-*Ph*_3_, 13H).

### 2.5. Film Preparation and LC Cell Assembly

Solutions of P4TB, P3TB, P2TB, PCUM, and PTRI were prepared in THF (1 wt.%). The solutions were filtered using a poly(tetrafluoroethylene) membrane with a pore size of 0.45 μm. Polymer thin films were prepared by spin coating (2000 rpm, 90 s) on a glass substrate. The LC cells were fabricated by assembling two polymer films on a glass slide using a 4.25 μm-thick spacer. The cells were filled with nematic LC, MLC-6608. The fabricated LC cells were sealed with epoxy glue.

### 2.6. Instrumentation

Various techniques were used for the characterization of the synthesized materials. ^1^H nuclear magnetic resonance (^1^H NMR) spectroscopy was performed on an MR400 DD2 NMR spectrometer (Agilent Technologies, Inc., Santa Clara, CA, USA); differential scanning calorimetry (DSC) was performed on a Q-10 (TA Instruments, Inc., New Castle, DE, USA) calorimeter; polarized optical microscopy (POM) images of the LC cells were acquired on a Nikon Eclipse E600 POL (NIKON, Inc., Tokyo, Japan) instrument equipped with a polarizer and Nikon Coolpix 995 digital camera (NIKON, Inc., Tokyo, Japan). The static contact angles of water on the polymer films were determined using a Krüss DSA10 (KRÜSS Scientific Instruments Inc., Hamburg, Germany) contact angle analyzer equipped with a drop shape analysis software (KRÜSS Scientific Instruments Inc., Hamburg, Germany). Surface energy values were calculated using Owens–Wendt’s equation as follows:

(1) $$\gamma_{sl}=\gamma_{s}+\gamma_{l}-2{\left(\gamma_{s}{}^{d}\gamma_{l}{}^{d}\right)}^{1/2}-2{\left(\gamma_{s}{}^{p}\gamma_{l}{}^{p}\right)}^{1/2}$$

where *γ_l_* is the surface energy of the liquid, *γ_sl_* is the interfacial energy of the solid–liquid interface, *γ_s_* is the surface energy of the solid, *γ_l_^d^* and *γ_l_^p^* are known for the test liquids, and *γ_s_^d^* and *γ_s_^p^* can be calculated from the measured static contact angles [55]. The contact angles for each sample were measured at least four times for three independently fabricated films, and the average values were used. Ultraviolet (UV) stability test of the LC cells was conducted using a VL-6.LC lamp (λ_max_ = 365 nm, Vilber Lourmat, Paris, France) with intensities of 5, 10, and 15 J/cm^2^ to corroborate the reliability to apply severe environment. The exposure dose of irradiated UV light on the LC cells was measured with a UV detector using GT-513 (Giltron, Seoul, Korea).

## 3. Results and Discussion

**Figure 2:**
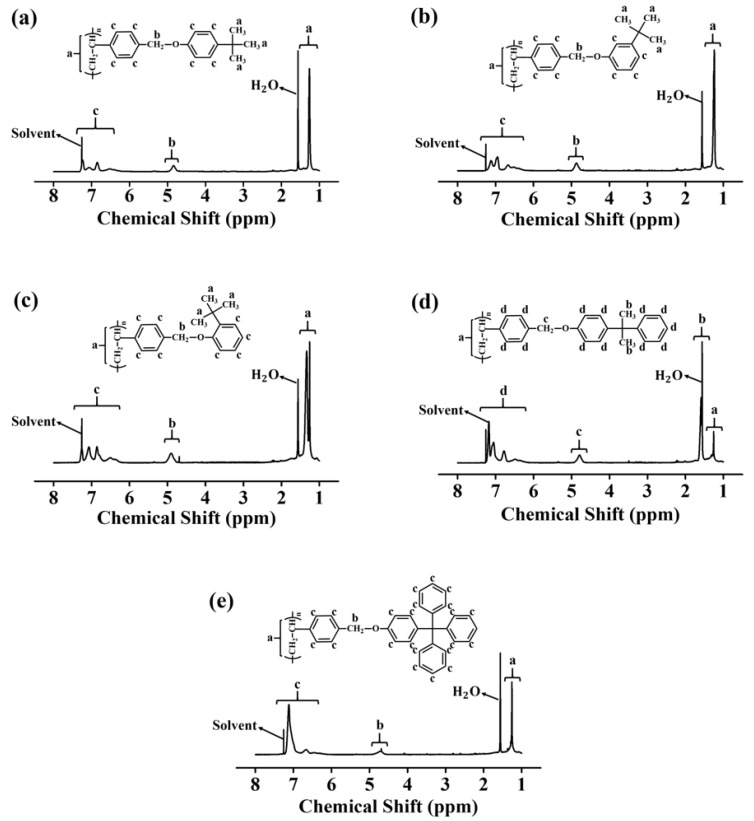
^1^H-nuclear magnetic resonance (^1^H NMR) spectrum of (**a**) P4TB, (**b**) P3TB, (**c**) P2TB, (**d**) PCUM, and (**e**) PTRI.

Figure 1 shows the synthetic routes to P2TB, P3TB, P4TB, PCUM, and PTRI. First, poly(4-chloromethylstyrene) was synthesized by the conventional free radical polymerization of 4-chloromethylstyrene with AIBN as the initiator under a nitrogen atmosphere. A series of polymers were obtained through modification reactions using a mixture of poly(4-chloromethylstyrene), phenol derivatives, and potassium carbonate in DMAc, a polar aprotic solvent. The characterization of P4TB is presented as a representative example. Almost complete conversion from chloromethyl to 4-(*tert*-butyl)-phenoxymethyl was obtained when 150 mol% of 4-(*tert*-butyl)phenol was used at 75 °C for 24 h, as evident from the ^1^H NMR spectra of the 4-(*tert*-butyl)phenol-containing homopolymer (Figure 2). The peaks at *δ* = 6.2–7.4 ppm (peak a) in the ^1^H NMR spectrum of P4TB correspond to the phenyl protons. The proton peaks from the *tert-*butyl side groups (*δ* = 0.9–1.5 ppm (peak c)) indicate the inclusion of tertiary carbon moieties in the polymer. Comparison of the integrated areas of the oxymethyl peak in the range 4.6–5.0 ppm and phenyl peaks in the range 6.2–7.4 ppm revealed that the degree of substitution from chloromethyl to oxymethyl was ~100%. Similar integrations and calculations for P3TB, P2TB, PCUM, and PTRI were performed, and the results were typically within ±10% of the values expected based on the synthesis. A comparison of the integrated peak areas of the oxymethyl peak and phenyl peaks revealed that the degree of substitution from chloromethyl to oxymethyl was ~100%. The high degree of substitution in this polymer modification reaction can be attributed to the electrophilicity of the benzylic carbon in poly(4-chloromethylstyrene) and to the structural stability of the phenolate anion as a nucleophile [53]. These polymers are soluble in many low boiling point solvents of medium polarities, such as THF and chloroform, and in polar aprotic solvents, such as *N,N*′-dimethylformamide, *N*-methyl-2-pyrrolidone, and *N,N*′-dimethylacetamide. The solubility of all the samples in various solvents is sufficient for use as thin-film materials.

**Figure 3:**
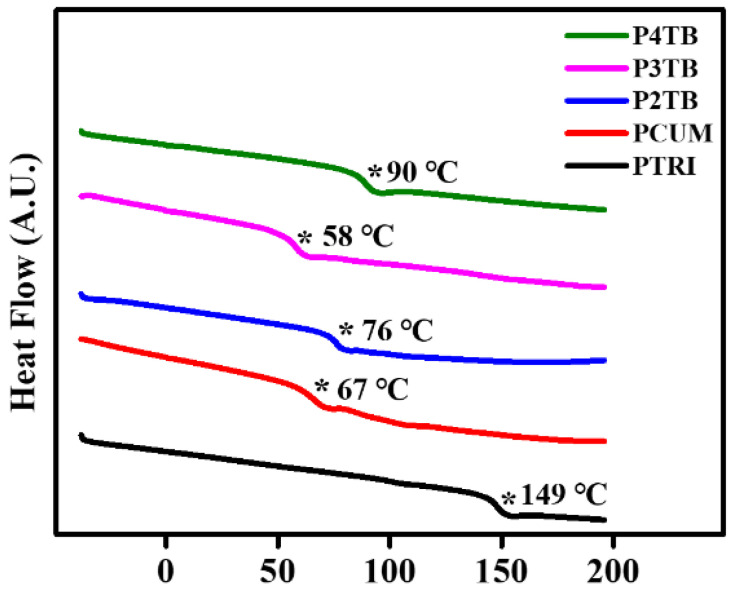
Differential scanning calorimetry (DSC) thermograms of PTRI, PCUM, P2TB, P3TB, and P4TB (* indicates the glass transition).

The thermal properties of the polymers were studied using DSC at a heating and cooling rate of 10 °C/min under a nitrogen atmosphere. All the polymers were amorphous, and only one glass transition was observed in the DSC thermograms. The glass transition temperatures were determined from the extrapolated intersection of the asymptotes to the glassy and rubbery regions for calculating the enthalpy, as illustrated in Figure 3. A decrease in *T_g_* of polystyrene derivatives having bulky substituents in the side group has been reported before [56]; for example, *T_g_* of P4TB, P3TB, P2TB, and PCUM is lower than that of polystyrene. However, the *T_g_* value increases from 104 °C for PCMS to 149 °C for PTRI. The *T_g_* values of P#TB decrease in the order P4TB > P2TB > P3TB. As expected, the *T_g_* value of P4TB with a *tert*-butyl group attached to the *para* position of the phenoxy group of the polystyrene side group is higher than those of P2TB and P3TB with *ortho-* and *meta*-type attachments, respectively [57]. The decrease in the *T_g_* values of P2TB and P3TB can be attributed to the kinking in the molecular chain due to the *ortho* and *meta* linkages, which increases the free volume of the polymer [57]. In addition, the *ortho* linkage increases the steric hindrance and decreases the flexibility, leading to higher *T_g_* values than those corresponding to the *meta* linkage [58]. The *T_g_* values of the polystyrene derivatives synthesized with 4-cumylphenol and 4-tritylphenol decrease in the order PTRI > P4TB > PCUM. The decrease in the *T_g_* value of PCUM with increasing phenyl ring of the bulky side groups has been previously reported and is ascribed to the increase in the free volume of the polymer because polymers having larger free volume have lower *T_g_* values [56]. However, as the number of phenyl rings of the terminal moiety in the side group increases from 1 to 3, the *T_g_* value increases from 67 °C for PCUM to 149 °C for PTRI. The increase in the *T_g_* value of PTRI can be attributed to the increased molecular interactions, such as *π*–*π* and van der Waals interactions, in the side groups [59]. 

**Figure 4:**
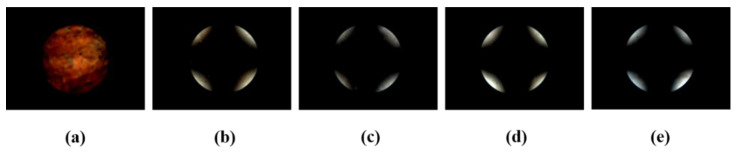
Conoscopic POM images of the LC cells fabricated with P4TB films under the following a weight ratio of the P4TB; (**a**) 0.001, (**b**) 0.01, (**c**) 0.05, (**d**) 0.1, and (**e**) 1.0 wt.%.

It is known that the molecular orientation of LCs can be affected by the chemical composition of the orientation layer, owing to the molecular interactions at the interface between LC molecules and the orientation layer. The orientations of LC molecules in the cells fabricated with polystyrene derivatives and grafted with #-(*tert*-butyl)-phenoxymethyl (including 4-(*tert*-butyl)-, 3-(*tert*-butyl)-, and 2-(*tert*-butyl)-phenoxymethyl moieties), 4-cumyl-phenoxymethyl, and 4-trityl-phenoxymethyl moieties were observed to systematically investigate the LC alignment behavior according to the position and structure of the terminal moieties. Figure 4 shows the conoscopic POM images of the LC cells fabricated from P4TB films onto glass substrates at P4TB weight concentrations of 0.001, 0.01, 0.05, 0.1, and 1.0 wt.%. Initially, a random planar alignment was observed for a P4TB weight ratio of less than 0.001 wt.% (Figure 4a). When the P4TB weight ratios were more than 0.01 wt.%, vertical alignment was observed, as evident from the Maltese cross pattern (Figure 4b–e). Therefore, 1 wt.% was selected as the optimum concentration of the coating solution for fabricating LC cells using P4TB, P3TB, P2TB, PCUM, and PTRI films, as previously reported [60].

**Figure 5:**
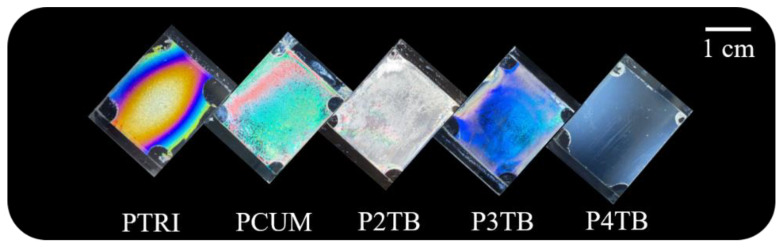
Photograph images of LC cells made from PTRI, PCUM, P2TB, P3TB, and P4TB films.

Photographic images of the LC cells made from P4TB, P3TB, P2TB, PCUM, and PTRI films are shown in Figure 5. The vertical LC alignment in the LC cells fabricated from P4TB films was considerably uniform over the entire area and was maintained for at least several months, whereas LC cells fabricated from P3TB, P2TB, PCUM, and PTRI films showed planar LC alignment. The effect of the position and structure of the terminal moieties in the side groups on the LC alignment behavior was investigated based on the POM images of the LC cells made from PTRI, PCUM, P2TB, P3TB, and P4TB films.

**Figure 6:**
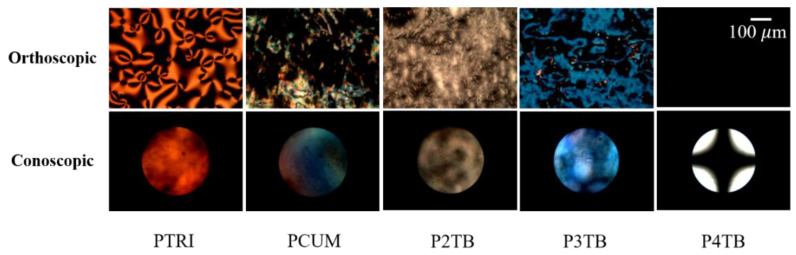
Orthoscopic (**top**) and conoscopic (**bottom**) POM images of LC cells made from PTRI, PCUM, P2TB, P3TB, and P4TB films.

Furthermore, the orthoscopic and conoscopic POM images of LC cells fabricated from these polymers were studied (Figure 6) for a more accurate analysis of the LC orientation behavior. The conoscopic POM images of LC cells fabricated with PTRI, PCUM, P2TB, and P3TB films revealed a planar LC alignment. On the other hand, the dark orthoscopic POM images and the Maltese cross pattern in the conoscopic POM image confirmed the vertical LC alignment of LC cells fabricated using the P4TB film. 

**Figure 7:**
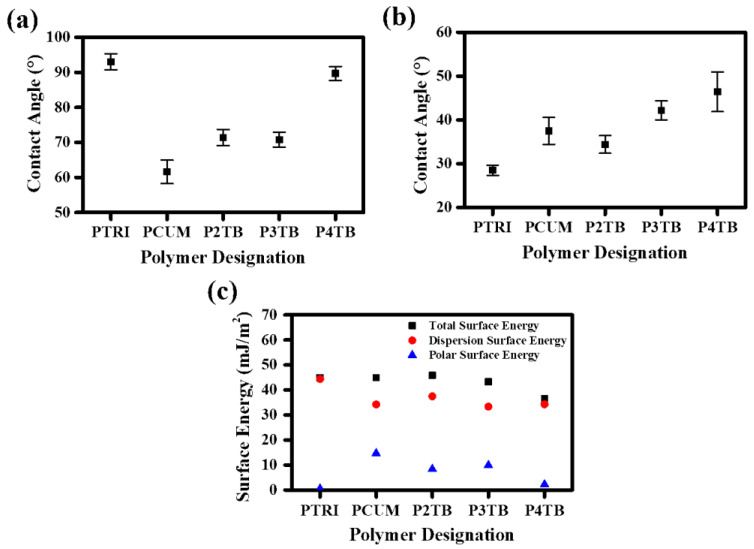
(**a**) Water, (**b**) diiodomethane contact angle, and (**c**) surface energy values of PTRI, PCUM, P2TB, P3TB, and P4TB films.

According to the photograph and POM images, only the P4TB film provided a stable uniform vertical orientation layer. The vertical LC alignment is related to the surface energy of the alignment layer surface and/or the steric repulsion between LC molecules and the alignment layer [61]. For example, nonpolar and bulky polyimide derivatives such as pentylcyclohexylbenzene [62] and 4-(*n*-octyloxy)phenyloxy [63] show vertical alignment behavior. Therefore, we attempted to analyze the LC alignment behavior of the P#TB, PCUM, and PTRI films with different positions and structures of the terminal moieties using several surface characterization techniques, including surface energy measurement of the polymer films. Figure 7 and Table 1 provide the surface energy values obtained by measuring the static contact angle of water and diiodomethane. The total surface energy was calculated using Owens–Wendt’s equation, which is a summation of the polar and dispersion contributions [55]. We also found that the vertical LC alignment could be affected by the critical surface energy of the polymer films. The total surface energy of P4TB exhibiting vertical LC alignment is lower than 36.6 mJ/m^2^, whereas P3TB, P2TB, PCUM, and PTRI, with a total surface energy of higher than 43.3 mJ/m^2^, do not show vertical LC alignment. These results indicate that *para*-type attachment of the *tert*-butyl side groups induced vertical LC alignment behavior, whereas *ortho*- and *meta*-type attachments of the same group could not. Different types of possible steric repulsions and/or interactions between LC molecules and the surfaces of the P4TB, P3TB, and P2TB films result in different LC alignment behaviors. PCUM and PTRI have additional phenyl groups in the terminal moieties, and the surface energies of these polymers are relatively high because the additional phenyl side groups increase the aromatic ring–aromatic ring interactions between LC molecules and polymer surfaces [64]. Therefore, the ability of P4TB to exhibit vertical alignment can be attributed to the increased steric repulsion between LC molecules and the polymer surfaces due to the incorporation of the nonpolar and bulky tertiary butyl moieties into the *para*-type attachment of the polystyrene side groups and the low surface energy (<36.6 mJ/m^2^) resulting from the unique chemical structure of the nonpolar carbon group.

**Figure 8:**
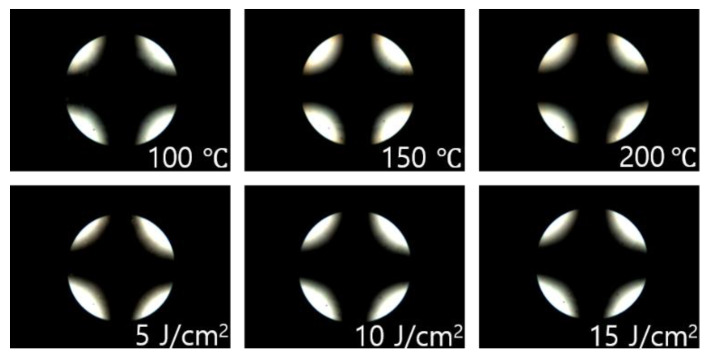
Concoscopic POM images of the LC cells made P4TB films, after thermal treatment at 100, 150, and 200 °C for 10 min and UV treatment at 5, 10, and 15 J/cm^2^, respectively.

The reliability of LC cells fabricated using the polymer films was investigated through a stability test of the LC alignment under severe conditions such as high temperatures and UV energies. Thermal and UV stabilities of the LC cell fabricated using the P4TB film were measured from the POM images obtained after heating at 100, 150, and 200 °C for 10 min and UV irradiation at 5, 10, and 15 J/cm^2^, respectively. As shown in Figure 8, no distinct differences in the vertical LC orientation on the P4TB films were observed through the Maltese cross pattern in the conoscopic POM images, indicating that the vertical LC orientation was maintained in the P4TB LC cell even at a high temperature and UV energy. For the P4TB films, the total surface energy obtained from the static contact angle of water and diiodomethane was also measured after heating and UV irradiation. The total surface energy, a characteristic of the P4TB film, was maintained in the range of 36–37 mJ/m^2^ even when the temperature and UV energies were increased to 200 °C and 15 J/cm^2^, respectively. Therefore, based on these results, P4TB, which exhibits thermal and UV stabilities, is a potential candidate as next-generation LC alignment films for diverse applications.

## 4. Conclusions

A series of #-(*tert*-butyl)-phenoxymethyl-substituted polystyrenes (P#TB, where # indicates the position of the *tert-*butyl group with respect to the oxymethyl group in disubstituted benzenes), 4-cumyl-phenoxymethyl-substituted polystyrene (PCUM), and 4-trityl-phenoxymethyl-substituted polystyrene (PTRI) were synthesized, and the liquid crystal (LC) alignment behavior of these films was investigated. The LC alignment behavior can be influenced by the position and structure of the tertiary carbon moiety attached to the phenoxy units in the side group of polystyrene. For example, LC cells fabricated using the P4TB film having tertiary butyl moieties as a *para*-type attachment to the phenoxy groups of polystyrene exhibited vertical LC alignment, whereas LC cells prepared using P3TB and P2TB films having tertiary butyl moieties as *meta*- and *ortho*-type attachments, respectively, to the phenoxy groups of polystyrene exhibited planar LC alignment. LC cells prepared from the PCUM and PTRI films with one and three phenyl rings, respectively, in the terminal moieties on polystyrene, exhibited planar LC alignment owing to the *π*–*π* and van der Waals interactions between LC molecules and the alignment layer. The vertical LC alignment was well correlated to the steric repulsion between LC molecules and polymer surfaces due to the nonpolar and bulky moieties attached to the side groups of the polymer and to the total surface energy of the polymer being lower than 36.6 mJ/m^2^. This provides the basic information for designing an LC alignment layer based on polymer films containing different terminal moieties.

## Figures and Tables

**Table 1:** Surface energy values and LC alignment properties.

Polymer Designation	Contact Angle (°) *^a^*		Surface Energy (mJ/m^2^) *^b^*			Vertical LC Aligning Ability *^c^*
	Water	Diiodo Methane	Polar	Dispersion	Total	
PTRI	93.0	28.5	0.6	44.4	45.0	X
PCUM	61.6	37.5	14.7	34.2	48.9	X
P2TB	71.4	34.4	8.4	37.6	46.0	X
P3TB	70.8	42.2	9.9	33.4	43.3	X
P4TB	89.7	46.4	2.3	34.3	36.6	O

*^a^* Measured from static contact angles. *^b^* Calculated from Owens–Wendt’s equation. *^c^* Circle (O) and cross (X) indicate polymer film have vertical and random planar, tilted LC aligning ability, respectively.

## Data Availability

The data presented in this study are available on request from the corresponding author.
